# Recent advances in targeting obesity, with a focus on TGF-β signaling and vagus nerve innervation

**DOI:** 10.1186/s42234-025-00172-x

**Published:** 2025-04-30

**Authors:** Sahara John, Krishanu Bhowmick, Andrew Park, Hai Huang, Xiaochun Yang, Lopa Mishra

**Affiliations:** 1 Institute for Bioelectronic Medicine, Divisions of Gastroenterology and Hepatology, Department of Medicine, Feinstein Institutes for Medical Research, Northwell Health, Manhasset, NY 11030 USA; 2 Cold Spring Harbor Laboratory, Cold Spring Harbor, NY 11724 USA; 3 Feinstein Institutes for Medical Research, Northwell Health, Manhasset, NY 11030 USA; 4 Department of Surgery, George Washington University, Washington, DC 20037 USA

**Keywords:** Obesity, TGF-β, Liver disease, Cancer, Vagus nerve stimulation

## Abstract

Over a third of the global population is affected by obesity, fatty liver disease (Metabolic Dysfunction-Associated Steatotic Liver Disease, MASLD), and its severe form, MASH (Metabolic Dysfunction-Associated Steatohepatitis), which can ultimately progress to hepatocellular carcinoma (HCC). Recent advancements include therapeutics such as glucagon-like peptide 1 (GLP-1) agonists and neural/vagal modulation strategies for these disorders. Among the many pathways regulating these conditions, emerging insights into transforming growth factor-β (TGF-β) signaling highlight potential future targets through its role in pathophysiological processes such as adipogenesis, inflammation, and fibrosis. Vagus nerve innervation in the gastrointestinal tract is involved in satiety regulation and energy homeostasis, and vagus nerve stimulation has been applied in weight loss and diabetes. This review explores clinical trials in obesity, novel therapeutic targets, and the role of TGF-β signaling and vagus nerve modulation in obesity-related liver diseases and HCC.

## Introduction

Obesity is a global epidemic affecting over a billion adults and children worldwide (WHO 2018). Approximately 75.27% of obese individuals develop metabolic dysfunction-associated steatotic liver disease (MASLD), while 33.67% progress to metabolic dysfunction-associated steatohepatitis (MASH) (Quek et al. 2023). Obesity is also linked to 65–78% of hypertension cases (Garrison et al. 1987), 75% of diabetes cases (Cioana et al. 2022), and 40% of cancers (CDC 2023). Moreover, every 1 kg/m² increase in body mass index (BMI) in 70-year-old women is associated with a 36% higher risk of Alzheimer’s disease, while a 5 kg/m² increase in BMI corresponds to a 29% greater risk of coronary heart disease and a 31% increase in all-cause mortality (Berrington de Gonzalez et al., 2010; Bogers et al. 2007; Gustafson et al. 2003). The prevalence of obesity varies across ethnic groups, with African Americans having the highest rate (36.1%), followed by Hispanics (28.7%), Whites (24.5%), and Asians (7.1%) (Kirby et al. 2012). Men have a slightly higher prevalence of obesity (43.0%) compared to women (41.9%) (Diseases 2021). These alarming statistics underscore the urgent need to address the causes of obesity and develop innovative approaches to mitigate its associated diseases.

An imbalance between energy intake and expenditure results in excess energy stored as triglycerides in adipose tissue, ultimately leading to obesity (Hagberg and Spalding 2024). Adipose tissue is categorized into white adipose tissue (WAT) and brown adipose tissue (BAT). WAT primarily stores energy and regulates satiety through large lipid droplets, while BAT dissipates energy as heat via its mitochondria-rich structure, playing a crucial role in thermoregulation. Dysregulation of these tissues contributes to the pathology of obesity and its associated diseases. Multiple signaling pathways are implicated in obesity, offering potential therapeutic targets. Among them, the Mitogen-Activated Protein Kinase (MAPK) pathway plays dual roles by regulating both adipogenesis and inflammation (Bost et al. 2005; Lawan et al. 2018); AMP-Activated Protein Kinase (AMPK) Pathway that reduces obesity by inhibiting adipogenesis and promoting thermogenesis, particularly in BAT (Martinez de Morentin et al. 2014). The Transforming Growth Factor-β (TGF-β) Pathway exerts complex regulatory effects on adipogenesis, inflammation, and energy expenditure. TGF-β signaling promotes inflammation in WAT while modulating thermogenesis and BAT function, influencing the overall metabolic state (Yadav et al. 2011). Dysregulation of this pathway is implicated in the progression of MASLD, MASH, and hepatocellular carcinoma (HCC) (Chen et al. 2018; Wang et al. 2021; Yang et al. 2014, 2024). The autonomic nervous system (ANS), particularly the parasympathetic vagus nerve, plays a crucial role in the neural regulation of obesity. The vagus nerve transmits signals related to food ingestion to the central nervous system (CNS), helping to regulate satiety, gastric motility, and gastric emptying (Bai et al. 2019). Vagus nerve dysfunction, commonly seen in obesity, reduces its regulatory efficiency (Lee et al. 2012; Loper et al. 2021).

## Therapeutic advances

Recent FDA-approved drugs and clinical trials offer hope for addressing obesity and its related conditions (see Table 1). Glucagon-like peptide 1 (GLP-1) receptor agonist (GLP-1 RA) Semaglutide and Tirzepatide, dual GIP/GLP-1 receptor agonists, have shown significant efficacy in reducing body weight and improving glucose control (Jastreboff et al. 2022; Wilding et al. 2021). They are highly effective, with 15% and 20.9% baseline weight reduction rates. Other GLP-1 RA, such as Orforglipron (Wharton et al. 2023), Liraglutide (Pi-Sunyer et al. 2015), CagriSima (Frias et al. 2023), GIP/GLP-1/glucagon RA, such as Retatrutide (Jastreboff et al. 2023) also reduce body weight ~ 10-15%. For type 2 diabetes, Tirzepatide was superior to Semaglutide (Frias et al. 2021). Resmetirom improves MASH by activating thyroid hormone receptor-beta (THR-β), which promotes lipophagy and hepatic fatty acid β-oxidation, thereby reducing liver fat (Harrison et al. 2023). 29.9% of patients taking 100-mg Resmetirom show MASH resolution (Harrison et al. 2024). However, these drugs are challenged with long-term effectiveness, as most patients taking Semaglutide regain two-thirds of their lost weight after one year of withdrawal (Wilding et al. 2022). More than 30% of users also stop taking these drugs within the first month, and only 42% of users meet the 12-week definition of clinical success (Intelligence 2024). Furthermore, 13.4% of patients treated with Semaglutide and 17–21% of patients treated with Tirzepatide do not achieve body weight reductions of even 5%, highlighting the need for alternative therapeutics to treat obesity (Garvey et al. 2023; Wilding et al. 2021). Treatment with Efruxifermin over 24 weeks, a Fibroblast growth factor 21 (FGF21) analogue, improved liver fibrosis at least one stage in patients with F2 or F3 fibrosis (Harrison et al. 2023).

**Table 1:** Clinical trials for obesity and obesity-related diseases

Mechanism of Action	Product Name	Condition and Phase	Primary Endpoint(s)	Duration (wk)	Ref.
GLP-1receptor agonist	Semaglutide	Obesity, Type 2 Diabetes	Mean weight loss − 14.9% in the semaglutide group as compared with − 2.4% with placebo (*P* < 0.001)	68	(Wilding et al. 2021)
GIP and GLP-1 receptor agonist	Tirzepatide	Obesity, phase II	Weight loss − 15.0% in 5-mg doses, -19.5% in 10-mg doses, and − 20.9% in 15-mg doses and − 3.1% in placebo (*P* < 0.001); -20.9%, glucose tolerance, Resolution of MASH, and no worsening of fibrosis	72	(Jastreboff et al. 2022)
GIP and GLP-1 receptor agonist	Tirzepatide	Type 2 Diabetes, phase III	HbA (1c) decrease was − 2.01%, -2.24%, and − 2.30% in 5, 10, and 15 mg of tirzepatide, respectively, and − 1.86% in semaglutide, which is not a significant difference	40	(Frias et al. 2021)
GIP and GLP-1 receptor agonist	Tirzepatide	MASH, and F2-F3 fibrosis, phase II	Resolution of MASH without worsening of fibrosis was 10% in the placebo group, 44%, 56% and 62% in the 5, 10, 15-mg group (*P* < 0.001). Improvement of at least one fibrosis stage without worsening of MASH was 30% in the placebo group, 55%, 51% and 51% in the 5, 10, 15-mg group	52	(Loomba et al. 2024)
GLP-1 receptor agonist with Amylin analogue	CagriSema	Obesity, Type 2 Diabetes; phase II	Weight loss − 15.6%, greater than semaglutide (-5.1%) and cagrilintide (-8.1%); HbA (1c) decrease − 2.2%, greater than cagrilintide (-0.9%)	32	(Frias et al. 2023)
GLP-1 receptor agonist	Orforglipron	Obesity; pahse II	Weight loss at 24 week − 8.6% to -12.6% and at 36 week − 9.4% to -14.7%	36	(Wharton et al. 2023)
THR-β agonist	Resmetirom	MASH with F1B, F2, or F3 Liver Fibrosis; phase III	NASH resolution with no worsening of fibrosis was achieved in 25.9% of 80-mg and 29.9% of 100-mg group, as compared with 9.7% of placebo group (*P* < 0.001). Fibrosis improvement by at least one stage with no worsening of the NAFLD activity score was achieved in 24.2% of the 80-mg group and 25.9% of 100-mg group, as compared with 14.2% of placebo group (*P* < 0.001)	52	(Harrison et al. 2024; Harrison, Taub, Harrison et al. 2023a, b)
Dual glucagon/GLP-1 receptor agonist	Survodutide	MASH with F1B, F2, or F3 Liver Fibrosis; phase II	Improvement in MASH with no worsening of fibrosis occurred in 47% of 2.4-mg group, 62% of 4.8-mg group, and 43% of 6.0-mg group, as compared with 14% of placebo group (*P* < 0.001); Decrease liver fat content in 63% of 2.4-mg group, 67% of 4.8-mg group, 57% of 6.0-mg group, and 14% of placebo group; improvement in fibrosis by at least one stage occurred in 34%, 36%, 34%, and 22%, respectively	48	(Sanyal et al. 2024)
GIP, GLP-1, and Glucagon receptor agonist	Retatrutide	Obesity, Type 2 Diabetes, phase II	Weight loss − 8.7% in the 1-mg group, -17.1% in the combined 4-mg group, -22.8% in the combined 8-mg group, and − 24.2% in the 12-mg group, as compared with − 2.1% in the placebo group. In the diabetes trial, with a decrease HbA (1c) -2.02% and weight loss up to -16.94%	48 (Obesity), 36 (Diabetes)	(Jastreboff et al. 2023) (Rosenstock et al. 2023)
FGF21 analogue	Efruxifermin	MASH and F2-F3 fibrosis, pahse IIb	Improvement in fibrosis of at least 1 stage and no worsening of NASH, at week 24, 15 (36%) of 42 in the 28 mg group (*p* = 0.033) and 14 (33%) of 43 in the 50 mg group (*p* = 0.123) versus eight (19%) of 43 patients in the placebo group met this endpoint	96	(Harrison et al. 2023)
pan-PPAR agonist	Lanifibranor	MASH, pahse IIb	A decrease of at least 2 points in the SAF-A score without worsening of fibrosis is higher in the 1200-mg dose than placebo (1200-mg dose vs. placebo, 55% vs. 33%); also a decrease in ALT, AST, etc.	24	(Francque et al. 2021)
GLP-1 receptor agonist	Liraglutide	Obesity, phase III	Weight loss a mean of 8.4 kg in liraglutide group, and 2.8 kg in placebo group	56	(Pi-Sunyer et al. 2015)
Pancreatic Lipase inhibitor	Orlistat	Obesity, phase NA	Weight reduction at year 1 end more than the placebo group (10.2% [10.3 kg] vs. 6.1% [6.1 kg]; at year 2 end patients switched from placebo to orlistat lost an additional 0.9 kg, compared with a mean regain of 2.5 kg in patients who continued on placebo	104	(Sjostrom et al. 1998)
Opioid antagonist and norepinephrine reuptake inhibitor	Bupropion / Naltrexone	Obesity, phase III	Weight loss − 6.1% in the naltrexone 32 mg plus bupropion group and − 5.0% in the naltrexone 16 mg plus bupropion group	56	(Greenway et al. 2010)
Norepinephrine-releasing agent and GABA receptor modulator	Phentermine / Topiramate	Obesity, phase III	Weight reduction − 8.1 kg and − 10.2 kg in the phentermine 7.5 mg plus topiramate 46.0 mg, and phentermine 15.0 mg plus topiramate 92.0 mg, respectively	56	(Gadde et al. 2011)
MC4R agonist	Setmelanotide	Obesity, phase II	Average reduction in BMI of 15%, a mean reduction in hunger score of 45%	16	(Roth et al. 2024)
Amylin analogue	Pramlintide acetate	Type 2 Diabetes, phase NA	A sustained reduction from baseline in HbA1c (− 0.68 and − 0.62% at weeks 26 and 52, respectively); a mean weight loss (− 1.4 kg vs. +0.7 kg with placebo at week 52)	52	(Hollander et al. 2003)
Biguanide	Metformin	Type 2 Diabetes, phase NA	Lowered fasting plasma glucose (19 mg/dL at 500 mg dosage to 78 mg/dL at 2000 mg dosage) and HbA1c (0.9% at 500 mg dosage to 2.0% at 2000 mg dosage)	14	(Garber et al. 1997)
ALK5 inhibitor	Galunisertib	Hepatocellular carcinoma, phase II	Combination of Galunisertib and sorafenib, the median OS Is 18.8 months, TGF-β1 responders (decrease of > 20% from baseline) vs. nonresponders have longer OS (22.8 vs. 12.0 months, *P* = 0.038)	> 104	(Kelley et al. 2019)
Bifunctional fusion protein (TGF-β trap/anti-PD-L1)	Bintrafusp alfa	Hepatocellular carcinoma, phase I	Median OS and PFS are 13.8 and 1.5 months in the dose-escalation cohort and 13.5 and 1.4 months in the dose-expansion cohort	180	(Lim et al. 2025)
TGF-β monoclonal antibody	SAR439459	Hepatocellular carcinoma and other cancers, phase I	Relatively safe and tolerable, discontinued due to the unclear efficacy and bleeding risk	52	(Baranda et al. 2024)
TGF-β monoclonal antibody	NI5793	Hepatocellular carcinoma and other cancers, phase I	No dose-limiting toxicities were observed	208	(Bauer et al. 2023)
αvβ1 inhibitor	PLN-1474	MASH; phase I, recruiting	-	-	(Slack et al. 2022)
ActRII antibody	Bimagrumab	Obesity, Type 2 Diabetes phase II	Weight loss − 6.5%	48	(Heymsfield et al. 2021)
GDF8/Activin A inhibitor	KER-065	Obesity, muscular dystrophy; phase I, recruiting	-	-	(Keros, 2024)
VNS, left cervical vagus	NCP model 101 stimulator	Obesity with depression, phase NA	At one-year average weight loss was 7 kg, at two-year loss of approximately 3.7 kg/year for individuals with an initial BMI of 32 kg/m^2^	104	
Anterior gastric wall	Transcend implantable gastric stimulator	Obesity, LOSS trail, phase NA	EWL was: 8.6% at 1 month, 15.8% at 3 months, 17.8% at 6 months, 21.0% at 10 months, and 21.0% at 15 months.	65	(De Luca et al. 2004)
Anterior gastric wall	Transcend implantable gastric stimulator	Obesity, SHAPE trail, phase NA	Control group lost 11.7% of excess weight and the treatment group lost 11.8% (*P* = 0.717)	52	(Shikora et al. 2009)
VNS, gastroesophageal junction	vBloc (Maestro Rechargeable System)	Obesity, ReCharege trail, phase NA	The mean EWL at 24 months was 21% (8% of body weight loss)	104	(Apovian et al. 2017; Ikramuddin et al. 2014; Shikora et al. 2015)
VNS, gastroesophageal junction	vBloc (Maestro Rechargeable System)	Obesity, EMPOWER trail, phase NA	At 12-month, EWL was 17 ± 2% for the treated and 16 ± 2% for the control group	52	(Sarr et al. 2012)
VNS, gastroesophageal junction	vBloc (Maestro Rechargeable System)	Type 2 Diabetes, Obesity, phase NA	At 12-month, EWL was 25 ± 4% (*P* < 0.0001), HbA1c declined 1.0 ± 0.2% (*P* = 0.02)	52	(Shikora et al. 2013)
VNS, greater occipital nerves	Multiprogram Trial Stimulator System	Obesity, a pilot study	Average body mass decrease of 8.7 kg	8	(Sobocki et al. 2013)
Anterior gastric wall	Gastric Electrical Stimulation	Obesity, a pilot study	The mean weight loss in individuals with a mean BMI of 40.8 ± 0.7 kg/m^2^ was 14.2 ± 4.5%	52–720	(Cigaina 2002)
Gastroesophageal junction	Transcend Implantable Gastric Stimulator	Obesity, a pilot study	Excess BMI lost was 30 ± 24% or 16 ± 12 Kg	36	(D’Argent 2002)
Anterior gastric wall	Gastric Electrical Stimulation	Obesity, phase NA	Excess weight lost 11.8% ± 17.6%, but no difference compared to control group	52	(Shikora et al. 2009)
VNS, transcutaneous auricular	Huatuo Ear vagus nerve stimulator	Type 2 Diabetes, a pilot study	Decreased glucose tolerance from 9.7 mmol/L to 7.5 mmol/L	12	(Huang et al. 2014)
PENS of dermatome T7	PC Neuromodulation System	Type 2 Diabetes, Obesity, phase NA	Decrease in glycemic levels of 62.1 mg/dL.	12	(Ruiz-Tovar et al. 2015)

Obesity not only contributes to metabolic disorders such as MASLD and MASH but also elevates the risk of cancers, particularly hepatocellular carcinoma (HCC). Chronic inflammation and lipid accumulation in the liver, driven by obesity, activate TGF-β signaling pathways, promoting hepatic fibrosis and metabolic dysregulation. Emerging therapies, such as Resmetirom (Rezdiffra), are designed to target these pathways and offer promising treatment options for MASH (Harrison et al. 2024; Harrison, Taub, Harrison et al. 2023a, b; Kokkorakis et al. 2024). Obesity-associated neural and metabolic dysfunction exacerbates these processes, establishing TGF-β as a critical link between obesity and cancer. Experimental mouse models with TGF-β signaling deficiencies shed light on its role in regulating obesity, MASLD, MASH, and HCC. For example, TGF-β receptor knockout in adipose or hepatic tissue leads to reduced inflammation, enhanced thermogenesis, and resistance to obesity-induced liver diseases (Wankhade et al. 2018; Yang et al. 2014). Neutralizing antibodies against the activin receptor-like kinase 7 (ALK7), induced a significant loss of adipose mass and improved insulin resistance in genetic and diet-induced obesity mouse models (Zhao et al. 2023). Blocking activin type II receptors (ActRII) signaling by monoclonal antibody Bimagrumab combined with GLP-1 RA Semaglutide can reduce fat mass while potentially preserving lean mass- although this remains controversial (Nunn et al. 2024).

**Fig. 1:**
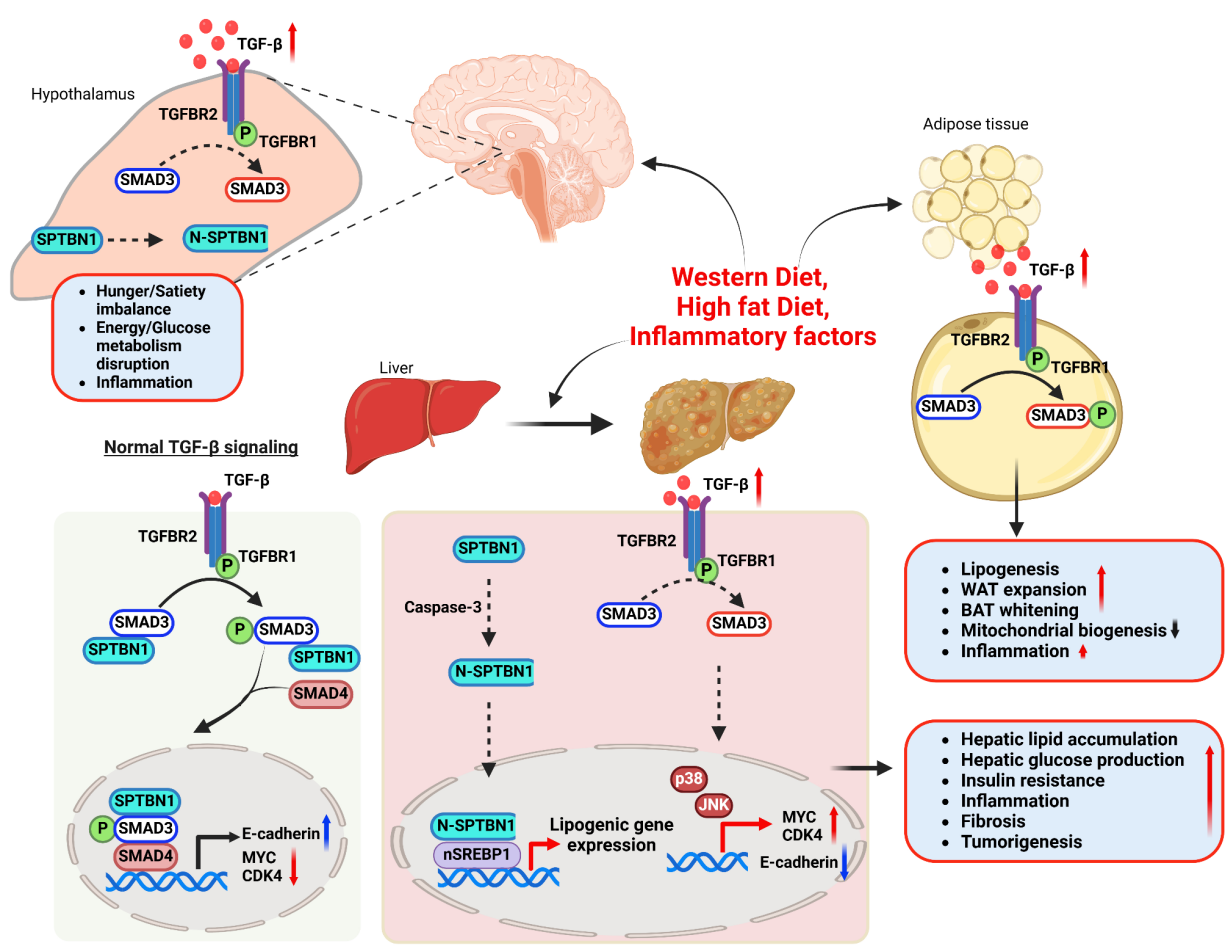
TGF-β signaling in obesity and related diseases. Overnutrition, Western diet, and increased circulating Fatty acids and glucose induces TGF-β expression and hypothalamic inflammation. Elevated TGF-β signaling interacts with other inflammatory pathways, promoting hypothalamic inflammation, which disturbs hunger and satiety signaling and disrupts energy balance. Under normal conditions (left), the TGF-β/Smad3/SPTBN1 pathway maintains lipid and energy homeostasis in the liver, preventing obesity, fibrosis, and cancer by directly suppressing key regulators like CDK4 and Myc. However, excessive energy intake, particularly from a Western diet, triggers Caspase-3-mediated cleavage of SPTBN1, impairing its interaction with SMAD3. This disruption promotes hepatic injury, lipogenesis, and oncogenic transformation, increasing susceptibility to metabolic syndrome and cancer. In adipose tissue (top right), elevated TGF-β levels drive lipogenesis, expansion of white adipose tissue (WAT), whitening of brown adipose tissue (BAT), and suppression of mitochondrial biogenesis. These changes contribute to heightened inflammation, exacerbating metabolic disturbances

Emerging non-invasive therapies, such as ultrasound stimulation of the vagus nerve, have shown promise in improving metabolic outcomes, including weight reduction, glucose homeostasis, and inflammatory modulation (Cotero et al. 2022; Huerta et al. 2021). In ageing septic rats, ghrelin/growth hormone treatment reverses immunosuppression by inhibiting the production of TGF-β through the vagus nerve (Zhou et al. 2020). Additionally, crosstalk between TGF-β signaling and neural pathways is evident by neural circuits that control appetite and energy balance modulated by TGF-β signaling in the hypothalamus and brainstem (Mendes et al. 2018; Yan et al. 2014) (Fig. 1). Disruptions in TGF-β signaling in these regions can lead to hyperphagia and obesity. This review explores the intersection of TGF-β signaling, neural regulation, and metabolic disorders in the context of obesity, MASLD, MASH, and HCC. Advances in mouse models and human trials will continue to inform strategies for modulating TGF-β and neural pathways, offering hope for treatments targeting the metabolic and oncogenic consequences of obesity.

## Mechanisms of obesity

WAT releases the adipokine leptin, which regulates food intake, energy expenditure, and sympathetic innervation, while its deficiency impairs thermogenesis and lipolysis, contributing to poor fat metabolism and severe obesity (Wang et al. 2020). WAT also releases proinflammatory cytokine tumor necrosis factor (TNF)-α, which influences hyperinsulinemia in obesity, and proinflammatory cytokine interleukin (IL)-6, which produces free fatty acids (FFAs), causing poor glucose and liver metabolism (Hotamisligil et al. 1995; Luan et al. 2023; Wueest and Konrad 2018). BAT expresses uncoupling protein 1 (UCP1), which uncouples the electron transfer chain from ATP synthesis to generate heat. Thermogenic fat is critical to adapting to cold temperatures and protects against obesity and metabolic dysfunction (Gomez-Hernandez et al. 2016; Valenzuela et al. 2023). BAT also releases chemokine C-X-C motif chemokine ligand-14 (CXCL14), which recruits M2 macrophages to WAT, promoting browning (Villarroya et al. 2019). Of the multiple signaling pathways involved in obesity, the mitogen-activated protein kinase (MAPK) signaling members, extracellular signal-regulated kinase 1/2 (ERK1/2) and c-Jun N-terminal kinase (JNK), promote obesity by enhancing inflammation, insulin resistance, and adipogenesis. At the same time, p38 MAPK inhibits obesity by increasing thermogenesis and activating BAT (Lawan et al. 2018; Solinas and Becattini 2017; Wen et al. 2022). Skeletal muscle knockout of mitogen-activated protein kinase phosphatase 1 (MKP1-MKO) raises both p38 MAPK and JNK phosphorylation. At 16 weeks of HDF feeding, MKP1-MKO mice weigh ∼20% less, with marked decreases in liver weight and hepatic triglyceride accumulation than their littermate controls. Consistent with this phenotype, MKP1-MKO livers exhibit significantly decreased expression of lipogenic genes peroxisome proliferator-activated receptor gamma (*PPARG)* and sterol regulatory element binding proteins 1c (*SREBP1C)* (Lawan et al. 2018). Similarly, dysregulated phosphatidylinositol 3-kinase (PI3K)/AKT signaling is associated with obesity and insulin resistance (Li et al. 2017; Savova et al. 2021). Interestingly, human phosphatase and tensin homolog (PTEN) mutations that lead to decreased PTEN expression increase the risk of obesity (Pal et al. 2012).

## Pathways involved in obesity

AMPK activation reduces eukaryotic translation initiation factor 2α (eIF2α) and SREBP-1 levels, reducing adipogenesis (Desjardins and Steinberg 2018; Garcia and Shaw 2017; Martinez de Morentin et al. 2014). Overexpression of constitutively active AMPK in the ventromedial hypothalamus reverses the weight loss in ovariectomy rats treated with estradiol. It is also associated with reduced UCP1, peroxisome proliferator-activated receptor-γ coactivator 1-α (PGC1α), and PGC1β expression in the BAT (Martinez de Morentin et al. 2014). SREBP-1 promotes lipogenesis and the release of FFAs, resulting in obesity. However, its activity is inhibited by bile acids, Farnesoid X receptor (FXR) agonists, and FGF19 analogs, preventing lipogenesis (Clifford et al. 2021; Zhou et al. 2017). FGF21 also reduces hepatic steatosis by inhibiting lipogenesis and increasing energy expenditure, insulin sensitivity (Xu et al. 2009).

Among the significant signaling pathways implicated in fibrosis and obesity and its associated diseases, the transforming growth factor-β (TGF-β) pathway has garnered recent attention due to its complex role in adipogenesis, inflammation, and energy expenditure. Obese humans and mice exhibit elevated TGF-β levels, with up to a 5-fold increase of TGF-β expression in obese mice. Blocking TGF-β signaling genetically (SMAD3^−/−^) and pharmacologically (anti-TGF-β antibody, 1D11) in mice lead to protective effects: decreased body weight gain and fat mass, improved insulin sensitivity, ameliorated hepatic steatosis. Additionally, almost 40% of HCC samples have somatic mutations in at least one gene whose product is a member of the TGF-β signaling pathway (Chen et al. 2018; Waddell et al. 2015).

## TGF-β signaling: overview

The TGF-β signaling pathway governs many cellular processes, including proliferation, differentiation, apoptosis, and extracellular matrix (ECM) production (Tan et al. 2012; Wrana et al. 1994). This pathway is initiated when TGF-β ligands (TGF-β1, TGF-β2, and TGF-β3) bind to TGF-β receptor types I and II, activating SMAD proteins that modulate gene expression (Feng and Derynck 1997; Massague 2012). Inhibitory SMADs, such as SMAD6 and SMAD7, counter this process by preventing receptor-regulated SMAD (R-SMAD) phosphorylation (Imamura et al. 1997; Kamiya et al. 2010). Beyond its SMAD-dependent mechanism, TGF-β also signals through SMAD-independent pathways. The TGF-β/Nodal subfamily encompasses TGF-β1, TGF-β2, TGF-β3, Nodal, Activins, growth differentiation factors (GDFs), Inhibin (which counteracts activin receptors), and Lefty1 and Lefty2 (which inhibit Nodal co-receptors). The bone morphogenic protein (BMP) subfamily includes BMPs, GDFs, anti-Mullerian hormone (AMH), and BMP3, a BMP receptor antagonist (Massague and Sheppard 2023). While this review centers on SMAD-dependent pathways, SMAD-independent signaling also contributes to obesity-related metabolic disorders (Fig. 1).

## TGF-β ligands in obesity

### TGF-β1

Elevated TGF-β1 levels are detected in the adipose tissue of obese humans and mice, and TGF-β1 inhibition confers protection against obesity (Alessi et al. 2000; Samad et al. 1997; Yadav et al. 2011) (Fig. 1). Inhibiting TGF-β1 disrupts Smad3 signaling, enhancing PGC1α activity, which induces mitochondrial biogenesis, UCP1 expression, and a lean phenotype in mice (Yadav et al. 2011). Insulin, induced by feeding, stimulates TGF-β1 expression in adipocytes, activating the TGF-β-SMAD3 pathway. This cascade promotes ECM remodeling, focal adhesion kinase (FAK)-AKT signaling, and adipocyte lipogenesis (Toyoda et al. 2022). Interestingly, the adipose-specific deletion of Interleukin-17 receptor C (IL-17RC) reduces TGF-β1 levels, impairing sympathetic innervation in BAT. Restoring TGF-β1 rescues innervation, underscoring its interaction with immune cells in BAT (Hu et al. 2020).

In the liver, elevated hepatic TGF-β1 promotes gluconeogenesis via adenosine 3′5′-cyclic monophosphate (cAMP)-dependent protein kinase-mediated forkhead box O (FoxO) 1 phosphorylation at serine 273, disrupting energy balance in obese, insulin-resistant mice (Pan et al. 2023) (Fig. 1). Elevated TGF-β1 in diabetic patients also promotes hepatic stellate cell (HSC) activation and gluconeogenesis (Sakurai et al. 2022). TGF-β1 knockout (KO) mice exhibit reduced adipose tissue formation and liver lipid accumulation alongside improved metabolic and liver function (Lee et al. 2023). Conversely, cardiac-derived TGF-β1 shields against weight gain and glucose intolerance by mitigating adipose inflammation and enhancing fatty acid oxidation (Longenecker et al. 2021).

### TGF-β2

Exercise induces the second ligand, TGF-β2 via lactate signaling, which enhances glucose and fatty acid metabolism in adipose tissue (Takahashi et al. 2019). TGF-β2 treatment reverses glucose intolerance and improves metabolic parameters in high fat diet (HFD)-fed mice, while its inhibition reduces mitochondrial respiration in human adipocytes. Restoring TGF-β2 maintains UCP1 expression during adipogenesis, underscoring its role in metabolic regulation (Halbgebauer et al. 2021). However, the study also reported hepatic TGF-β2 upregulation correlates with fibrosis and HCC progression in mouse models and cell lines (Dropmann et al. 2016).

### TGF-β3

The third ligand-TGF-β3 levels rise in white adipose tissue (WAT) during obesity, stimulating adipocyte precursor proliferation (Petrus et al. 2018). Mice lacking Kruppel-like factor (KLF) 10 in CD4^+^ T cells show decreased TGF-β3 secretion, impaired regulatory T-cell (Treg) migration, and develop obesity, insulin resistance, and fatty liver. Transferring wild-type CD4^+^ Tregs reverses these effects, highlighting TGF-β3’s regulatory role in immune-metabolic interactions (Wara et al. 2020).

## TGF-β receptors in obesity

### TGF-βRI

Deletion of TGF-βRI in adipose tissue fosters beige adipogenesis (Wankhade et al. 2018). Pharmacological inhibition of TGF-βRI (e.g., RepSox) promotes brown adipogenesis via UCP1 upregulation, preventing obesity (Tu et al. 2019). TGF-βRI inhibition also mitigates MASLD progression, with agents such as Isoquercetin reducing fibrosis and inflammation (Qin et al. 2018). Conversely, TGF-βRI upregulation exacerbates MASLD and fibrosis, as seen in models of SIX1 (Sine oculis homeobox homologue 1) or SHMT2 (Serine hydroxymethyl transferase 2) expression (Gao et al. 2023a; Y. Gao et al., b).

### TGF-βRII

Similarly, TGF-βRII promotes obesity and its hepatic complications. TGF-βRII deletion improves thermogenic gene expression and reduces HFD-induced adiposity and resolved MASH (Yang et al. 2014; Zhao et al. 2022). Hepatic miRNA let-7b-5p is a potential reason why TGF-βRII is pro-fibrotic, as let-7b-5p is much higher in MASLD patients when TGF-β signaling is enhanced, whereas let-7b-5p is reduced in TGF-βRII KO in hepatocytes (Zhao et al. 2022).

### SMAD signaling in obesity

SMAD proteins regulate lipid metabolism and inflammation but demonstrate complex roles. Overexpression of SMAD2/3/4 improved, whereas overexpression of SMAD7 worsened obesity-associated metabolic syndromes in HFD-fed obese mice (Seong et al. 2018). Conversely, adipocyte deletion of SMAD3 protects mice from obesity, diabetes, and hepatic steatosis (Yadav et al. 2011). SMAD3 also contributes to insulin resistance and type 2 diabetes. SMAD3 represses insulin gene transcription and impairs β-cell function, while its deficiency improves glucose tolerance, insulin sensitivity, and diabetic kidney injuries (Lin et al. 2009; Sun et al. 2015; Wang et al. 2022).

### SMAD3 adaptor proteins

Adaptor proteins in the TGF-β pathway, such as βII-Spectrin, Smad anchor for receptor activation (SARA), and Disabled-2 (Dab2), play critical roles in facilitating SMAD protein activation and regulation (Mishra and Marshall 2006; Penheiter et al. 2010). Among them, βII-Spectrin, encoded by the SPTBN1 gene, is a key adaptor for SMAD3. It promotes SMAD3 activation by TGF-β receptors and interacts with SMAD3 in the nucleus to regulate gene transcription (Fig. 1). Elevated levels of SPTBN1 and Caspase-3 have been observed in both obese humans and mice, linking SPTBN1 to obesity pathogenesis (Rao et al. 2021).

### New targets: SMAD3 adaptor SPTBN1

Liver-specific knockout of SPTBN1 in mice protects against obesity, providing a potential therapeutic avenue for metabolic disorders (Rao et al. 2021). HFD and Western diet (WD) induce Caspase-3-mediated cleavage of SPTBN1, and the resulting fragments translocate to the nucleus, driving the expression of pro-obesity genes (Rao et al. 2021). Therapeutic strategies targeting Caspase-3 cleavage of SPTBN1 or modulating nuclear translocation of SPTBN1 cleavage products could mitigate obesity-related metabolic dysregulation (Fig. 1).

In cancer, particularly hepatocellular carcinoma (HCC), high SPTBN1 expression is associated with poor prognosis, positioning it as a potential biomarker and therapeutic target (Rao et al. 2021). SPTBN1 regulates p65 levels, suppressing inflammatory cytokine expression and thereby reducing inflammation and immune suppression, hallmarks of liver cancer (Lin et al. 2021). SPTBN1 also interacts with suppressor of variegation 3-9-enhancer of zeste-trithorax domain containing lysine methyltransferase (SETD) 7, which methylates Yes-associated protein (YAP), a key regulator of the Hippo signaling pathway and tumorigenesis. This interaction enhances autophagy in hepatic stem cells, underscoring its significance in maintaining cellular homeostasis and preventing malignant transformation (Chen et al. 2022). This leads to multiple future therapeutically relevant opportunities: **Obesity Treatment**: Modulating SPTBN1 expression or function could provide a novel therapeutic strategy to combat obesity by inhibiting the Caspase-3–SPTBN1 axis and its downstream effects. **Cancer Therapy**: Blocking toxic SPTBN1 cleavage or enhancing its regulatory interactions (e.g., with SETD7 or autophagy pathways) could suppress HCC progression. Therapeutics targeting SPTBN1’s role in Wnt signaling or its interactions with YAP might also limit tumorigenesis. **Inflammation Control**: SPTBN1-mediated regulation of inflammatory pathways positions it as a dual target for treating metabolic disorders and cancer by reducing systemic and local inflammation. Targeting βII-Spectrin (SPTBN1) in a tissue-specific manner offers a promising avenue for managing obesity and its complications, including progression to liver diseases and HCC (Rao et al. 2021; Yang et al. 2024). Continued research into its molecular interactions and regulatory networks will refine its potential as a therapeutic target.

### BMPs

BMP4 promotes WAT browning and reduces WAT mass, improving metabolic health in obese models (Modica et al. 2016; Qian et al. 2013; Son et al. 2011). Liver-specific BMP4 expression reduces lipid accumulation and MASLD progression via the mammalian target of rapamycin complex (mTORC) 1 pathway (Peng et al. 2019).

### GDFs

GDF15 improves metabolic parameters, including insulin sensitivity and glycemic control, in obesity models via glial cell line-derived neurotrophic factor (GDNF) family receptor α–like (GFRAL) receptor signaling (Sjoberg et al. 2023; Wang et al. 2023; Yang et al. 2017). Conversely, elevated GDF8 (myostatin) exacerbates muscle loss and metabolic dysfunction, presenting a therapeutic target for obesity-related sarcopenia (Hittel et al. 2009; McPherron and Lee 1997; Rebbapragada et al. 2003). Furthermore, small molecule inhibitors of GDF8/Activin (KER-065) and antibodies of its receptor ActRII (Bimagrumab) have shown promising results in promoting weight loss while preserving muscle mass in both animal models and clinical trials (Heymsfield et al. 2021; Keros, 2024).

### Activins

Activin A maintains adipocyte progenitor populations and promotes obesity via macrophage-mediated secretion (Zaragosi et al. 2010). Neutralizing Activin A decreases cell proliferation and adipocyte differentiation, revealing potential for therapeutic interventions.

## Targeting TGF-β signaling to alleviate obesity

Despite significant advances in understanding the role of the TGF-β pathway in obesity, obesity-induced diseases, and cancer, critical questions remain about whether this pathway functions predominantly as a driver or suppressor in these conditions. Addressing this dual role is essential for designing effective therapeutic interventions. Leveraging new technologies can provide deeper insights into how obesity-related metabolic changes influence disease progression and cancer development.

## Advances in imaging technologies

Recent breakthroughs in microscopy have revolutionized the study of TGF-β signaling. For instance, Single-molecule fluorescence Imaging and Tracking (SMIT) enables real-time observation of individual SMAD3 molecules docking at cell membranes and being activated by TGF-βRI (Li et al. 2016). Stochastic Optical Reconstruction Microscopy (STORM) provides even higher resolution, making it an invaluable tool for elucidating intricate protein interactions in the TGF-β pathway (Xu et al. 2017). Cryo-electron microscopy (Cryo-EM) has revealed that integrin αvβ8 can bind to latent TGF-β (L-TGF-β) and activate TGF-β/SMAD signaling without releasing TGF-β from its latent complex, providing a novel perspective on pathway activation (Campbell et al. 2020).

## AI-Driven insights into TGF-β regulation

Artificial intelligence (AI) tools like AlphaFold have uncovered unexpected molecular interactions, such as the extensive interface between fibrillin and latent TGF-β binding protein 1 (LTBP1) in supporting TGF-β activation (Lockhart-Cairns et al. 2022). These findings open new avenues for therapeutic targeting, particularly in conditions where TGF-β activation is dysregulated.

## Limitations of current models and the promise of organoids

While mouse models have been invaluable for studying TGF-β signaling, they often fall short in replicating human-specific conditions (Table 2). Organoids, three-dimensional structures derived from stem cells or primary tissues, offer a promising alternative. These models closely mimic the architecture and functionality of human tissues, providing a more accurate platform for studying TGF-β signaling in obesity-related diseases. Knockdown of TGF-βRII in gastric organoids with co-occurring CDH1 (encodes protein E-cadherin) and TP53 mutations induces features of metastatic diffuse gastric cancer, including severe dysplasia, focal invasion, and robust in vivo tumorigenicity. This underscores the critical role of TGF-β signaling in tumor progression (Nadauld et al. 2014). Liver organoids mimicking MASLD have been established using three key factors: free fatty acids (FFAs), the genetic variant PNPLA3^I148M^, and APOB/MTTP mutations related to familial hypo-/non-β lipoproteinemia. These models are proving instrumental in drug screening and advancing our understanding of MASLD (Hendriks et al. 2023). Combining organoids with CRISPR-based screening has identified FADS2 (fatty acid desaturase 2) as a critical determinant of hepatic steatosis from a 35-gene lipid metabolism dataset. This highlights the potential of organoid models in uncovering gene functions in TGF-β signaling under obesity-induced metabolic conditions (Hendriks et al. 2023). The integration of advanced imaging techniques, AI-driven tools, and organoid technologies provides an unprecedented opportunity to unravel the complex dynamics of TGF-β signaling. These tools not only enhance our understanding of the pathway’s role in obesity and related diseases but also pave the way for personalized therapeutic strategies. For example, by leveraging these advanced methodologies, we can refine therapeutic approaches to mitigate obesity, its associated metabolic complications, and its progression to cancer.

**Table 2:** Mouse models for studying roles of TGF-β signaling in obesity-related diseases

Gene	Mouse Model	Model Design	Diet	Model Phenotype	Ref.
TGF-β1	TGF-β1^−/−^ (C57BL/6)	Disruption of TGF-β1 Exon 3 with a neomycin cassette	High-fat diet	Resistant to adipose tissue hypertrophy, liver steatosis, and insulin resistance (20 wks)	(Lee et al. 2023)
TGF-β3	TGF-β1^Lβ3/Lβ3^	Ligating PCR products of TGF-β1 LAP with sequence for the mature HA-tagged ligands of murine TGF-β3, murine TGF-β2, and porcine TGF-β1	High-fat diet	Weight loss and improved glucose tolerance (8 wks)	(Hall et al. 2013)
TGF-βRI	TGF-βRI^AdKO^ (C57BL/6)	TβRI floxed mice intercrossed with aP2-Cre mice	High-fat diet	Weight loss and glucose intolerance (24 wks)	(Wankhade et al. 2018)
TGF-βRII	TGF-βRII^ΔHEP^ (C57BL/6)	Albumin-Cre recombinase transgenic mice intercrossed with Tgfbr2^flox/flox^ mice	High-fat diet	Lower MASLD scores and reduce liver weight (16 wks)	(Zhao et al. 2022)
SMAD2	β-cell-specific SMAD2^−/−^ (C57BL/6J)	Ins1cre mice intercrossed with SMAD2fx/fx	High-fat diet	Improved glucose tolerance, insulin secretion, and insulin sensitivity (12 wks)	(Saleh et al. 2021)
SMAD3	SMAD3^−/−^ (C57BL/6J and Lep ob/ob mice)	Disruption of SMAD3 exon 8 with neomycin cassette	High-fat diet	Weight loss and insulin resistance (10 wks)	(Yadav et al. 2011)
SMAD3	SMAD3^−/−^ (C57BL/6J)	SMAD3 heterozygous mice intercrossed	High-fat diet	Weight loss and insulin resistance (18 wks)	(Tan et al. 2011)
SPTBN1	SPTBN1^LSKO^ (C57BL/6J)	Disruption of exons 24 to 26 of Sptbn1, with neomycin cassette. Neo cassettes removed by intercrossing with Flp mice. Sptbn1-Flox mice intercrossed with Albumin-Cre.	High-fat diet and Western diet	Resistant to obesity, MASLD, and MASH	(Rao et al. 2021)
SPTBN1	Aldh2^−/−^SPTBN1^+/−^ (C57BL/6Jx129SvEv)	Aldh2^−/−^ mice intercrossed with Sptbn1^+/−^ mice	Western diet	Metabolic syndrome, Obesity, MASH	(Yang et al. 2024)

## Limitations of targeting TGF-β signaling

The high expression of L-TGF-β isoform in many tissue types of healthy individuals results in limited effectiveness by antibodies and other biologics targeting TGF-β isoforms. Given the critical and pleiotropic roles that TGF-β signaling plays in normal development, tissue homeostasis, and immunomodulation, broad inhibition of TGF-β signaling often results in unacceptable toxicity. Preclinical studies have shown pan-inhibition TGF-β (monoclonal antibodies or small molecule inhibitor) to cause multiple organs damage, including cardiovascular toxicity (degeneration/necrosis and inflammation in the aortic root), hemorrhage in the GI tract and abdomen, and abnormal wound healing (Mitra et al. 2020; Stauber et al. 2014). Currently, only a few drugs targeting TGF-β pathway are under clinical trials. Integrin αvβ1 inhibitor (a small molecule, PLN-1474) is in a Phase 1 trial for MASH fibrosis (Slack et al. 2022) and GDF8/Activin A inhibitor (K065) is in a Phase 1 trial for obesity and muscular dystrophy (Keros, 2024). Other drugs targeting TGF-β pathway have been withdrawn because their preclinical effects did not translate to humans or due to the occurrence of clinical adverse events (Baranda et al. 2024; Kossen 2019; Smith et al. 2024). Improving our understanding and monitoring of adverse effects are essential factors for successfully inhibiting this key pathway. Most of all, targeting specific aspects through, for example adaptor proteins such as SPTBN1 could limit such toxicities (Yang et al. 2024).

## The role of the autonomic nervous system in regulating energy balance and therapeutic implications

### Innervation of vagus nerve and energy homeostasis

**Fig. 2:**
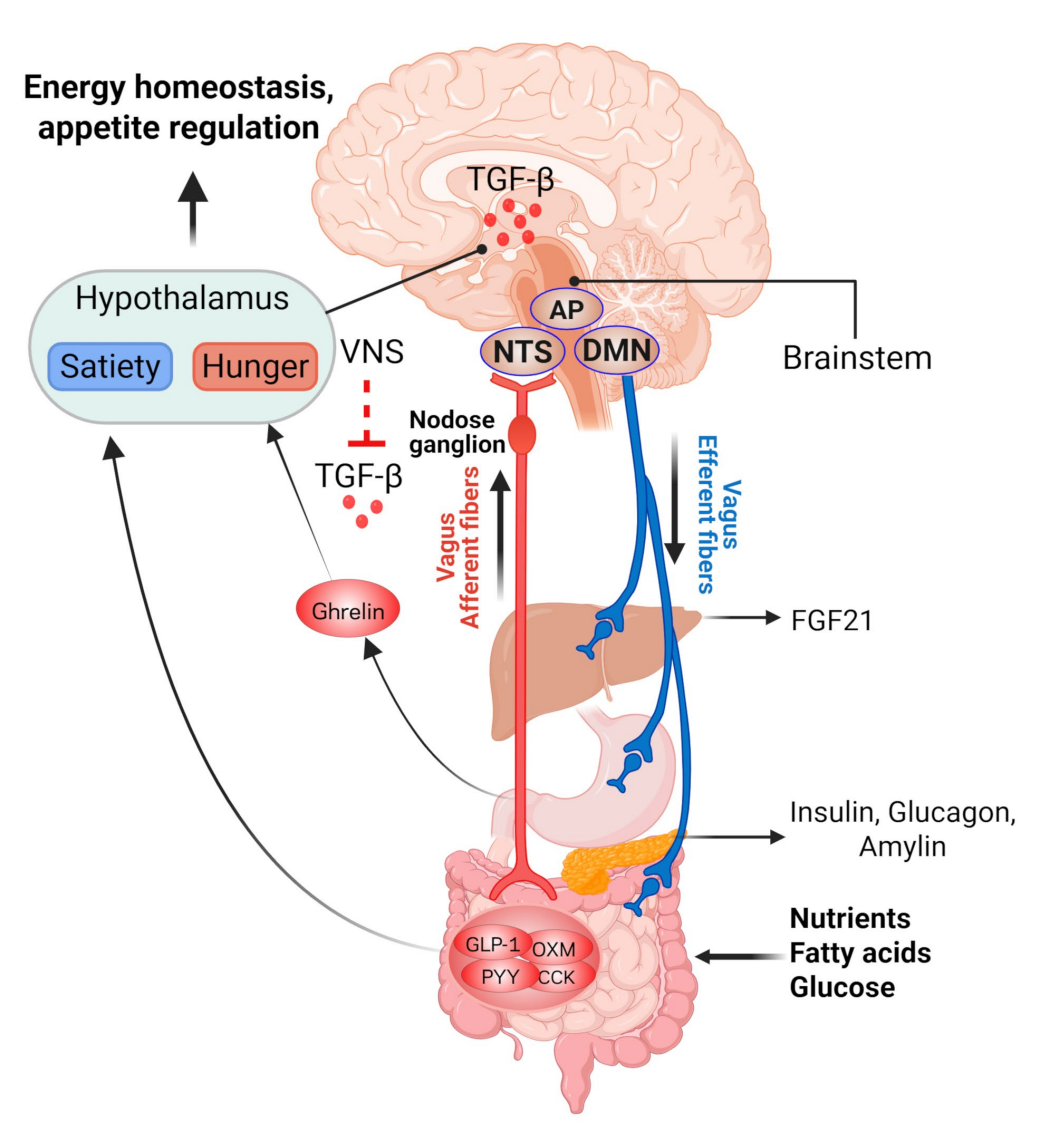
Vagus nerve innervation and regulation of satiety. Vagus nerve innervations of metabolic organs are depicted. Peripheral signals related to satiety and hunger signal reach the nucleus of the solitary tract (NTS) in the brainstem via afferent vagal nerves or via the circulation reach the median eminence of the hypothalamus and area postrema (AP) of the brainstem. The NTS then projects to the dorsal motor nucleus (DMN), which modulates intestinal motility, secretion, glucose production, and pancreatic secretion through efferent vagal nerves. peptide tyrosine tyrosine, PYY; glucagon-like peptide-1, GLP-1; oxyntomodulin, OXM; cholecystokinin, CCK; fibroblast growth factor 21, FGF21; gastrointestinal, GI

The autonomic nervous system (ANS) plays a pivotal role in maintaining energy balance by regulating both food intake and energy expenditure. Within the ANS, the parasympathetic nerve transmits critical information about food ingestion and digestion to the central nervous system (CNS), influencing satiety by modulating gastric motility, emptying, and gut hormone release (Bai et al. 2019). This regulatory feedback loop is essential for maintaining body weight homeostasis. The vagus nerve provides the gastrointestinal tract, pancreas, and liver parasympathetic innervation (Fox and Powley 1985) (Fig. 2). The vagus nerve contains approximately 80% sensory fibers (afferent) and 20% motor fibers (efferent). The afferent vagal pathways are likely the most crucial link between the gut and brain in modulating satiety signals. Vagal afferent neurons receive post-ingestive information from the GI tract through three primary mechanisms: mechanoreceptor stimulation in response to gastric distension, release of gut hormones (GLP-1, CCK, PYY et al.) triggered by the nutritional composition of consumed food, and the direct action of certain nutrients (e.g. short-chain fatty acids). Metabolic information also is conveyed via chemoreceptors located in the hepatoportal system (Yi et al. 2010). Signals from peripheral receptors travel through vagal afferents to the area postrema/nucleus of the solitary tract (AP/NTS) region in the brainstem, which integrates sensory input from the GI tract and abdominal viscera, as well as oral taste information. The NTS, in turn, projects back to the gut via vago-vagal reflexes through the dorsal motor nucleus (DMN) (Powley 2021) (Pavlov and Tracey 2012) (Fig. 2). Activation of this pathway regulates gut responses, including intestinal transit time, motility, absorption rates, and nutrient exposure of enteroendocrine cells. These processes influence the release of GI hormones and pancreatic secretions, ultimately playing a key role in satiety regulation. Disruptions in vagus nerve activity are commonly observed in metabolic disorders such as obesity and type 2 diabetes mellitus (T2DM) (Lee et al. 2012; Loper et al. 2021). Both high fat and carbohydrate diets impair vagal activity and disrupt satiety regulation (Loper et al. 2021). Reduced vagal tone in these conditions exacerbates metabolic dysregulation, prompting researchers to investigate novel non-pharmacologic strategies targeting the vagus nerve.

### Vagus nerve stimulation (VNS): A novel therapeutic frontier

Several studies have conceptualized treating obesity with the use of electrical stimulation of the stomach and performed initial animal and human studies in 1990’s (Cigaina et al. 1996, 1999), and confirmed that gastric electrical stimulation (GES) can effectively induce weight loss, with minimal disruption of physiology side effects of conventional bariatric procedures. Preliminary clinical trials have shown promising decreases in body weight and increases satiety by GES treatment (Cigaina 2002; D’Argent 2002). The actual mechanism of gastric stimulation has not yet been identified, data from animal and human studies have suggested potential mechanisms such as vagus nerve stimulation (VNS) (Burneo et al. 2002), fundic relaxation (Orthey et al. 2018), and ghrelin inhibition (De Luca et al. 2004). VNS has been approved for the treatment of refractory epilepsy since 1997 and the treatment of resistant depression later. Interestingly, these studies reported refractory epilepsy patients lost body weight after VNS treatment (Burneo et al. 2002). VNS treatment and weight loss in resistant depression patients were positively correlated with initial BMI; at one year, the average weight loss was 7 kg, and the BMI drop was 2 kg/m^2^ (Pardo et al. 2007). Emerging evidence highlights the therapeutic potential of vagus nerve therapy in addressing obesity and related metabolic disorders.

### Clinical trials of VNS in obesity related diseases

Multiple neuromodulation clinical trials for obesity and obesity related conditions have been conducted (Table 1). Currently, only vBloc (Maestro Rechargeable System) has been approved by FDA in 2015, other devices such as the Transcend^™^ implantable gastric stimulation (IGS) device (Shikora et al. 2009), and TANTALUS System (Sanmiguel et al. 2009) et al. are still being investigated. Different electric stimulation devices target various vagal areas: around the esophagogastric junction trunk by vBloc, anterior gastric wall by Transcend Implantable Gastric Stimulator, left cervical vagus by NCP model stimulator and auricular concha area vagus by Transcutaneous Electrical Nerve Stimulator. In European participants, the LOSS (Laparoscopic Obesity Stimulation Survey) study reported patients with gastric stimulation reached 21.0% excess weight loss (EWL) at 15 months (De Luca et al. 2004). The SHAPE (Screened Health Assessment and Pacer Evaluation) trial has shown that gastric stimulation did not decrease body weight (Shikora et al. 2009). The EMPOWER study revealed that at 12 months, vBloc treatment had a 17% EWL, not greater than 16% EWL in the control group (Sarr et al. 2012). Then, ReCharge (Maestro Rechargeable System for the Treatment of Obesity) trial reported vBloc at 12-month has a significant 24.4% EWL (9.2% of initial body weight loss) compared to 15.9% EWL (6.0% of initial body weight loss) in the control group (Ikramuddin et al. 2014), and sustained effects observed at 18–24 month (Apovian et al. 2017; Shikora et al. 2015), but not be viewed as clinically significant. In T2DM patients, vBloc therapy exhibited significant EWL (25 ± 4%, *P* < 0.0001), and HbA1c decrease (1.0 ± 0.2%, *P* = 0.02, baseline 7.8 ± 0.2%) at 12-month (Shikora et al. 2013). Transcutaneous auricular VNS (taVNS) via the ear vagus nerve stimulator have shown improved glucose tolerance (from 9.7 mmol/L to 7.5 mmol/L) in T2DM patients (Huang et al. 2014) (Table 1). So far, these electric stimulation trials have shown inconsistent results and far less weight loss than GLP-1 receptor agonists or bariatric surgery (Puzziferri et al. 2014). Therefore, further optimization is needed to increase the effectiveness of vagal modulation.

### Mechanisms of VNS in obesity treatment

In a rat model, a battery-free VNS device at the surface of the stomach reduced body weight by 38% compared to the control groups. This significant reduction underscores the efficacy of VNS in modulating food intake and energy expenditure (Yao et al., 2018). Another rat model revealed VNS increased the number of mast cells in the GI wall and c-Fos expression in nodosal ganglia. This demonstrates that VNS can increase vagal afferent satiety signals (Gil et al. 2009). VNS also altered the gut hormones releasing. A diet-induced obese rat model reveals that VNS treatment significantly increased plasma GLP-1 (72.9 ± 8.4 vs. 44.3 ± 5.9, *P* = 0.012, vs. Sham) and PYY (72.3 ± 7.8 vs. 36.9 ± 7.9, *P* = 0.008, vs. Sham) levels (Dai et al. 2020). Banni et al. previously showed that VNS increased rat plasma non-esterified fatty acids (NEFA), hepatic PPARα expression and its potential ligand N-palmitoylethanolamide (PEA) expression in mesenteric adipose tissue (Banni et al. 2012); these results indicate VNS may increase lipolysis in white adipose tissue and fatty acid oxidation in the liver. One clinical study suggested VNS intervention was significantly correlated to BAT activity ((*r* = 0.935, *P* < 0.001) and increased energy expenditure (Vijgen et al. 2013). Under vBloc stimulation, multiple genes (e.g., CCKβ receptor and Leptin receptor) expression changed in the brainstem and hippocampus. Still, no changes in gut hormones (e.g., glucagon and GLP-1) were observed (Johannessen et al. 2017). Thus, vBloc may activates vagal signaling to the brain while blocking vagal signaling to the gut, resulting in increased satiety, reduced food intake, and ultimately, weight loss. Therefore, brain, GI tracts, liver, and adipose tissue respond to vagal modulation. Understanding the multiple aspects of these above mechanisms is key to maximizing the benefits of VNS in the future.

### Development of non-invasive VNS

Ultrasound stimulation of the vagus nerve represents a novel, non-invasive approach to modulating neural and metabolic pathways in obesity. Among the promising techniques is peripheral focused ultrasound stimulation (pFUS), a representative method to activate vagus nerve pathways. Recent studies using pFUS targeting the porta hepatis demonstrated significant metabolic improvements in mouse models of obesity. An 8-week regimen of vagus nerve stimulation reduced Body weight and fat mass, Serum triglycerides (TG) and alanine aminotransferase (ALT) levels, Pro-inflammatory cytokines, such as tumor necrosis factor (TNF) and interleukin-1β (IL-1β). Additionally, pFUS improved glucose homeostasis in mice fed a Western diet, highlighting its potential to counteract diet-induced metabolic disturbances (Cotero et al. 2022; Huerta et al. 2021).

### Regulation of TGF-β signal via vagal activity

In a septic rat model, vagus nerve activity has been demonstrated to inhibit TGF-β production under hormone treatment. (Zhou et al. 2020). In the rat myocardial infarction model, pharmacologically preserving vagal activity by pyridostigmine improved cardiac diastolic function and collagen deposition via inhibition of TGF-β1 and TGF-β1-activated kinase expression. (Lu et al. 2014). In two heart failure models, low-level of transcutaneous VNS protects cardiac function by anti-inflammatory and antifibrotic effects and decreases TGF-β production and collagen deposition (Elkholey et al. 2022; Wang et al. 2014). Thus, vagal stimulation suppresses TGF-β1 expression. However, in a Crohn’s disease pilot trial, VNS restored a homeostatic vagal tone, and reduced inflammation via increase of anti-inflammatory TGF-β and decrease of proinflammatory factors (Sinniger et al. 2020). Therefore, regulating TGF-β expression by vagal activity may depend on the specific states of inflammation, fibrosis progression and is context dependent.

### Implications of VNS in obesity, MASLD, MASH, and cancer

Given the complex interplay between metabolic dysregulation and obesity-related diseases, including metabolic-associated steatotic liver disease (MASLD), metabolic-associated steatohepatitis (MASH), and cancer, VNS offers a promising avenue for therapeutic intervention: VNS directly addresses the dysregulated energy balance by reducing appetite, enhancing satiety, and promoting metabolic efficiency. These effects could reduce the burden of obesity-related comorbidities. By lowering circulating pro-inflammatory cytokines and improving liver function markers such as ALT, VNS may mitigate hepatic inflammation and fibrosis, critical drivers of MASLD and its progression to MASH. Chronic inflammation and obesity are well-established risk factors for cancer. By reducing systemic inflammation and promoting metabolic balance, VNS could play a role in lowering cancer risk in obese individuals.

### Advantages of vagus nerve stimulation

VNS offers several advantages over traditional pharmacologic interventions:

- Alternative Noninvasive VNS: the latest application of ultrasound in vagus nerve stimulation (e.g. pFUS) offers a non-invasive option at the sub-organ level, reducing the risk of complications associated with surgical.
- Targeted Mechanism: VNS directly modulates neural circuits involved in energy and metabolic regulation, providing a precise therapeutic effect.
- Multi-System Benefits: Beyond metabolic improvements, VNS may enhance gastrointestinal motility, liver function, and systemic inflammation, addressing the multifaceted consequences of obesity.

### Limitations of VNS trials

While VNS treatments are effective at improving obesity and its related metabolic disorders by stimulating the vagus nerve, these clinical trials also have their limitations.

- Placebo response issue. In VNS trials, ensuring a proper placebo control group is difficult because the participants can feel the stimulation effects of the VNS devices, which don’t provide true blinding. This leads to potential placebo responses that complicate the interpretation of results (Sarr et al. 2012).
- Low participants compared to clinical drug trials. VNS trials have fewer participants due to high expenses and invasive procedures (Pardo et al. 2007). This limits the statistical power of these studies, which makes it harder to generalize findings.
- Mild to moderate effects on weight loss. While VNS devices help with weight loss, these effects are usually mild to moderate compared to other standard interventions such as GLP-1 receptor agonists (Ozempic) or bariatric surgery (Fadel et al. 2023). This limits the widespread usage and adoption of VNS devices for treating obesity.
- Surgical risks and long-term compliance. Although VNS devices are laparoscopically implanted, surgical risks still pose a threat to patients, such as infection, device malfunction, and nerve damage. Furthermore, the VNS devices require battery replacements every 4–8 years, which places a physical and financial burden on patients (Vonck et al. 2005). Evidence also shows that VNS can directly affect ventricular function, particularly in cases of ventricular fibrillation (VF), which is responsible for a significant portion of sudden cardiac deaths (Al-Khatib and Stevenson 2018). In animal models, VNS increases the variability of the dominant VF frequency and decrease left ventricular wall motion (Naggar et al. 2014). Additionally, VNS significantly reduced blood pressure, potentially inducing ischemia in organs (Naggar et al. 2014).

The application of VNS for metabolic diseases is still in its early stages, but its potential is evident. Future research should focus on: Long-Term Efficacy and Safety: Evaluating the sustained effects of VNS on weight management and metabolic health in human clinical trials. Optimization of Stimulation Protocols: Determining the optimal parameters for stimulation frequency, duration, and anatomical targeting. Identifying patient-specific factors that predict responsiveness to VNS could help tailor treatments and maximize efficacy.

## Novel insights on synergistic strategies

Physical exercise can protect against several diseases, especially metabolism-associated disorders, and contribute to health maintenance. Recreational cyclists can suppress latent TGF-β1 activation and are associated with better lipid profiles (Eka Widiastuti et al. 2021). In skeletal muscle, increased TGF-β1 contributes to impaired exercise response by suppressing key mitochondrial regulators, such as PGC1α and AMPKα2 (Bohm et al. 2016). Therefore, one potential synergistic strategy may be combining the targeting of TGF-β pathway members or adaptor (e.g., Sptbn1) with physical exercise. As modulation of vagus nerve can downregulate TGF-β level (Elkholey et al. 2022; Go et al. 2022; Zhou et al. 2020), another synergistic strategy should be combining targeting TGF-β pathway with VNS. The efficacy of TGF-β signaling inhibition can potentially be enhanced through these combinations. Additionally, VNS can be targeted at the sub-organ level, allowing for more precise inhibition of TGF-β within specific organs rather than affecting the entire body. Specifically, in adipose tissue inhibition TGF-β signaling may suppress adipogenesis and lipogenesis. Targeting adipose tissue by VNS is difficult because WAT lacks significant vagal innervation (Giordano et al. 2006), but adipose tissue has been responsive to VNS indicating the possibility for vagal intervention (Banni et al. 2012). Combining VNS with lifestyle interventions, pharmacologic treatments, or emerging technologies (e.g., AI-driven monitoring systems) to enhance therapeutic outcomes is also a potential approach in the future.

## Conclusions

The autonomic nervous system, particularly the vagus nerve, represents a promising target for non-pharmacologic interventions in obesity and its complications. Techniques such as VNS show potential in reducing body weight, improving metabolic markers, and alleviating inflammation. Because the prevalence of obesity, MASLD, MASH, and obesity-related cancer continues to rise, integrating VNS into targeting TGF-β therapeutic strategies offers a cutting-edge approach to combat these interrelated conditions. This review explores the intersection of TGF-β signaling, neural regulation, and metabolic disorders in the context of obesity, MASLD, MASH, and HCC. Advances in mouse models and human trials will continue to inform strategies for modulating TGF-β and neural pathways, offering hope for specific treatments targeting the metabolic and oncogenic consequences of obesity.

## Data Availability

No datasets were generated or analysed during the current study.

## Abbreviations

AI: Artificial intelligence
ALK7: Activin receptor-like kinase 7
ALT: Alanine aminotransferase
AMH: Anti-Mullerian hormone
AMPK: AMP-Activated Protein Kinase
ANS: Autonomic nervous system
AP: Area postrema
BAT: Brown adipose tissue
BMI: Body mass index
BMP: Bone morphogenic protein
cAMP: Adenosine 3′5′-cyclic monophosphate
CCK: Cholecystokinin
CNS: Central nervous system
Cryo-EM: Cryo-electron microscopy
CXCL14: Chemokine C-X-C motif chemokine ligand-14
Dab2: Disabled-2
DMN: Dorsal motor nucleus
ECM: Extracellular matrix
eIF2α: Eukaryotic translation initiation factor 2α
ERK1/2: Extracellular signal-regulated kinase 1/2
EWL: Essential weight loss
FADS2: Fatty acid desaturase 2
FAK: Focal adhesion kinase
FGF21: Fibroblast growth factor 21
FFAs: Free fatty acids
FoxO1: Forkhead box O1
FXR: Farnesoid X receptor
GDFs: Growth differentiation factors
GES: Gastric Electrical Stimulation
GFRAL: GDNF family receptor α-like
GLP-1: Glucagon-like peptide 1
GLP-1 RA: GLP-1 receptor agonist
HCC: Hepatocellular carcinoma
HFD: High-fat diet
HSC: Hepatic stellate cell
IL-1β: Interleukin-1β
IL-17RC: Interleukin-17 receptor C
JNK: C-Jun N-terminal kinase
KLF10: Kruppel-like factor 10
KO: Knockout
LTBP1: Latent TGF-β binding protein 1
MAPK: Mitogen-activated protein kinase
MASLD: Metabolic-associated steatotic liver disease
MASH: Metabolic dysfunction-associated steatohepatitis
mTORC1: Mammalian target of rapamycin complex 1
NTS: Nucleus of the solitary tract
PENS: Percutaneous neurostimulation
pFUS: Peripheral focused ultrasound stimulation
PGC1α: Peroxisome proliferator-activated receptor-γ coactivator 1-α
PI3K: Phosphatidylinositol 3-kinase
PPARG: Peroxisome proliferator-activated receptor gamma
PTEN: Phosphatase and tensin homolog
PYY: Peptide tyrosine tyrosine
SARA: Smad anchor for receptor activation
SETD7: Suppressor of variegation 3-9-enhancer of zeste-trithorax domain containing lysine methyltransferase 7
SHMT2: Serine hydroxymethyl transferase 2
SIX1: Sine oculis homeobox homologue 1
SMIT: Single-molecule fluorescence imaging and tracking
SREBP1C: Sterol regulatory element binding proteins 1c
STORM: Stochastic optical reconstruction microscopy
TG: Triglycerides
TGF-β: Transforming growth factor-β
THR-β: Thyroid hormone receptor-beta
TNF: Tumor necrosis factor
T2DM: Type 2 diabetes mellitus
UCP1: Uncoupling protein 1
VNS: Vagus nerve stimulation
WAT: White adipose tissue
WD: Western diet
YAP: Yes-associated protein
